# Distinct patterns of SSR distribution in the *Arabidopsis thaliana *and rice genomes

**DOI:** 10.1186/gb-2006-7-2-r14

**Published:** 2006-02-21

**Authors:** Mark J Lawson, Liqing Zhang

**Affiliations:** 1 Department of Computer Science, Virginia Tech, 655 McBryde, Blacksburg, VA 24060, USA

## Abstract

**Background:**

Simple sequence repeats (SSRs) in DNA have been traditionally thought of as functionally unimportant and have been studied mainly as genetic markers. A recent handful of studies have shown, however, that SSRs in different positions of a gene can play important roles in determining protein function, genetic development, and regulation of gene expression. We have performed a detailed comparative study of the distribution of SSRs in the sequenced genomes of *Arabidopsis thaliana *and rice.

**Results:**

SSRs in different genic regions - 5'untranslated region (UTR), 3'UTR, exon, and intron - show distinct patterns of distribution both within and between the two genomes. Especially notable is the much higher density of SSRs in 5'UTRs compared to the other regions and a strong affinity towards trinucleotide repeats in these regions for both rice and *Arabidopsis*. On a genomic level, mononucleotide repeats are the most prevalent type of SSRs in *Arabidopsis *and trinucleotide repeats are the most prevalent type in rice. Both plants have the same most common mononucleotide (A/T) and dinucleotide (AT and AG) repeats, but have little in common for the other types of repeats.

**Conclusion:**

Our work provides insight into the evolution and distribution of SSRs in the two sequenced model plant genomes of monocots and dicots. Our analyses reveal that the distributions of SSRs appear highly non-random and vary a great deal in different regions of the genes in the genomes.

## Abstract 2

A comparative study of the distribution of single sequence repeats in rice and *Arabidopsis *reveals that the repeat patterns vary a lot in different genomic regions.

## Background

Simple sequence repeats (SSRs) are tandem repeat nucleotides (oftentimes defined as being between 1 and 6 base-pairs (bp)) in DNA sequences. They can be found in any genome (both eukaryote and prokaryote) and in any region (protein coding regions and non-coding regions). Historically, SSRs were used often as genetic markers, helping to classify and identify various species. However, recent research has shown that SSRs have many important functions in terms of development, gene regulation, and evolution.

The locations of SSRs appear to determine the types of functional role SSRs might play, and changes in SSRs in different genetic locations can lead to changes in the phenotypes of an organism [1]. SSRs in coding regions can determine whether or not a gene gets activated or whether the protein product is truncated [1]. For instance, expansion of CAG repeats in the coding region of *HD *genes in humans can lead to Huntington's disease, most likely through activation of so-called 'toxic' proteins. The development of the nervous system in *Drosophila *appears to be associated with length variation of trinucleotide repeats in genes involved in developmental control [2]. Most recently, Fondon and Garner [3] have shown that the fast morphological evolution in domesticated dogs is due to the contraction/expansion of SSRs in the coding regions of the *Alx-4 *and *Runx-2 *genes.

SSRs in other genic regions can have large effects on organisms as well. For example, SSRs in 5'-untranslated regions (UTRs) have an effect on gene transcription and/or regulation [1]. The human calmodulin-1 (*hCALM1*) gene has a CAG repeat in a 5'UTR that when deleted causes a decrease in expression by 45% [4]. Intron SSRs can affect gene transcription, regulation, mRNA splicing, and gene silencing [1]. For example, the first intron of the gene encoding tyrosine hydroxylase contains a TCAT repeat that acts as a transcription regulatory element [5]. SSRs found in 3'-UTRs are involved in gene silencing and transcription slippage [1], as in the case of a CTG expansion in a kinase gene that causes myotonic dystrophy type 1 through transcription slippage [6].

The functional study of SSRs has been largely restricted to animals. In plants, the majority of research used SSRs as genetic markers to study populations and genetic diversity [7-9] and to determine sex in dioecious plants [10]. Several studies have been done to characterize the distribution of SSRs in *Arabidopsis*. For example, Casacuberta *et al*. [11] examined the abundant types of mono- and dinucleotide repeats in coding sequences of the unfinished *Arabidopsis *genome. Zhang *et al*. [12] did a more comprehensive survey of SSRs in *Arabidopsis *and showed that SSRs in general were more favored in upstream regions of genes and that trinucleotide repeats were the most common repeats found in the coding regions. The purpose of this paper is to compare SSRs between the two plant species: *Arabidopsis thaliana *and rice (*Oryza sativa*). These two plants have their entire genomes largely sequenced, so in-depth comparisons of SSRs can be made for not only their entire genome, but specific regions as well, such as exons, introns, and UTRs.

## Results

The coding regions (exons) of 26,416 genes in *Arabidopsis thaliana *and 57,915 genes in rice were analyzed. The 57,915 rice genes include 14,273 transposable element (TE)-related genes. Excluding these genes from analyses does not change our results qualitatively, and, therefore, we report here only the results for the 57,915 genes. The 5'UTR regions of 16,355 genes and the 3'UTR regions of 17,617 genes were used for *Arabidopsis*. For rice these values were 12,907 and 14,839, respectively. The lower number of UTR sequences than coding regions is due to the fact that UTRs are curated only when there is full-length cDNA or expressed sequence tag (EST) evidence supporting the annotation [13]. Therefore, although the number of UTRs is reduced, we have high data quality. Finally, the intron data of 21,157 genes in *Arabidopsis *and 45,633 genes in rice were analyzed as well. The total lengths of these regions are shown in Table 1. Detailed in the following sections are the SSR amounts for the various regions. Tables 2 and 3 list the most common repeat types, including all circular permutations and their complements, similar to previous analyses done on this topic [12,14].

### Whole genome SSRs

The *Arabidopsis *genome contains a total of 104,102 SSRs (Table 1). The genome average SSR density is thus approximately 875 per mega-base (MB). SSRs with periods of 1 to 10 (mono-, di-, tri-, and so on) account for 33.9% (35,256 of 104,102), 12.8% (13,295), 17.5% (18,244), 5.5% (5,731), 7.8% (8,097), 8.9% (9,261), 5.2% (5,392), 3.5% (3,612), 3.6% (3,720), and 1.4% (1,494), respectively.

**Figure 1:**
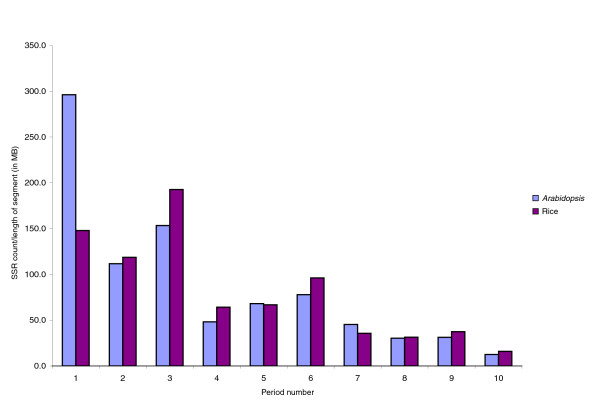
Comparison of whole genome SSR densities. A comparison of the SSR densities (for SSRs of period 1 to 10) in the whole genome of *Arabidopsis *and rice.

In comparison, the rice genome contains a total of 298,819 SSRs (Table 1). The genome average SSR density is approximately 807/MB. SSRs with periods of 1 to 10 account for 18.3% (54,809), 14.7% (43,949), 23.9% (71,373), 7.9% (23,756), 8.3% (24,718), 11.9% (35,602), 4.4% (13,216), 3.9% (11,628), 4.6% (13,854), and 2% (5,914), respectively. Figure 1 shows the corresponding SSR densities.

### Exon SSRs

The exon regions in *Arabidopsis *contain a total of 12,168 SSRs (Table 1). The average SSR density is thus approximately 334/MB. SSRs with periods 1 to 10 account for 1% (121), 2.2% (264), 65.4% (7,961), 1% (121), 2.4% (296), 17.3% (2,102), 1.6% (193), 1.6% (192), 6.9% (844), and 0.6% (74), respectively.

**Figure 2:**
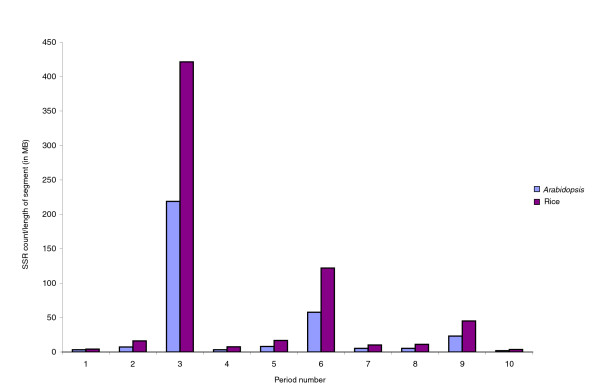
Comparison of exon SSR densities. A comparison of the SSR densities (for SSRs of period 1 to 10) in the coding (exon) regions of *Arabidopsis *and rice.

In comparison, the rice exon regions contain a total of 55,338 SSRs (Table 1). The average SSR density is approximately 658/MB. SSRs with periods of 1 to 10 account for 0.6% (354), 2.4% (1,353), 64% (35,437), 1.2% (637), 2.5% (1,409), 18.6% (10,268), 1.5% (856), 1.7% (929), 6.9% (3,791), and 0.5% (304), respectively. Figure 2 shows the corresponding SSR densities.

### Intron SSRs

The intron regions in *Arabidopsis *contain a total of 17,756 SSRs (Table 1). The average SSR density is approximately 815/MB. SSRs with periods of 1 to 10 account for 39.5% (7,011), 15.1% (2,674), 11.2% (1,981), 7.2% (1,280), 8.1% (1,432), 7.9% (1,410), 4.5% (805), 3% (538), 2.4% (421), and 1.1% (204), respectively.

**Figure 3:**
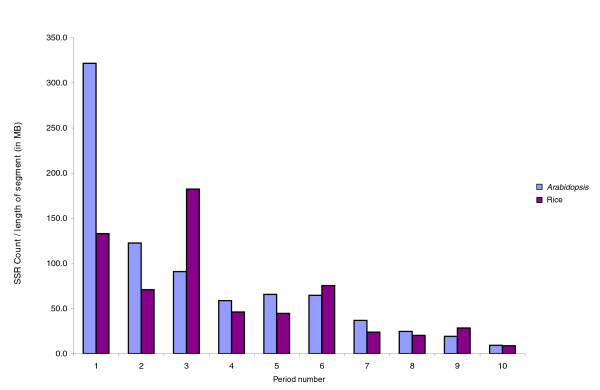
Comparison of intron SSR densities. A comparison of the SSR densities (for SSRs of period 1 to 10) in the intron regions of *Arabidopsis *and rice.

In comparison, the rice intron regions contain a total of 87,529 SSRs (Table 1). The average SSR density is approximately 634/MB. SSRs with periods of 1 to 10 account for 21% (18,360), 11.2% (9,794), 28.8% (25,169), 7.3% (6,379), 7% (6,160), 11.9% (10,410), 3.8% (3,297), 3.2% (2,805), 4.5% (3,932), and 1.4% (1,223), respectively. Figure 3 shows the corresponding SSR densities.

### 5'UTR SSRs

The 5'UTR regions in *Arabidopsis *contain a total of 6,146 SSRs (Table 1). The average SSR density is approximately 2,364/MB. SSRs with periods of 1 to 10 account for 18.4% (1,130), 28.1% (1,727), 29.7% (1,827), 4.5% (275), 5% (310), 6.8% (417), 2.6% (160), 2.2% (137), 1.8% (110), and 0.9% (53), respectively.

**Figure 4:**
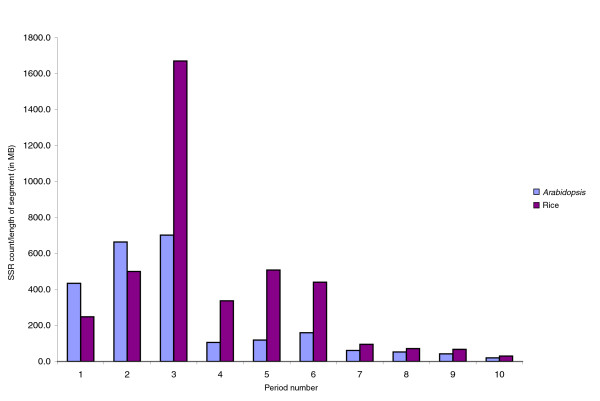
Comparison of 5'-UTR SSR densities. A comparison of the SSR densities (for SSRs of period 1 to 10) in the 5'-UTR regions of *Arabidopsis *and rice.

In comparison, the rice 5'UTR regions contain a total of 12,310 SSRs (Table 1). The average SSR density is approximately 3971/MB. SSRs with periods of 1 to 10 account for 6.2% (769), 12.6% (1,552), 42.1% (5,179), 8.5% (1,046), 12.8% (1,575), 11.1% (1,367), 2.4% (297), 1.8% (222), 1.7% (209), 0.8% (94), respectively. Figure 4 shows the corresponding SSR densities.

### 3'UTR SSRs

The 3'UTR regions in *Arabidopsis *contain a total of 4,910 SSRs (Table 1). The average SSR density is approximately 982/MB. SSRs with periods of 1 to 10 account for 34.6% (1,700), 12.5% (615), 17.5% (858), 8.8% (434), 8.4% (411), 7.8% (383), 4.2% (208), 3.3% (160), 2.2% (107), 0.7% (34), respectively.

**Figure 5:**
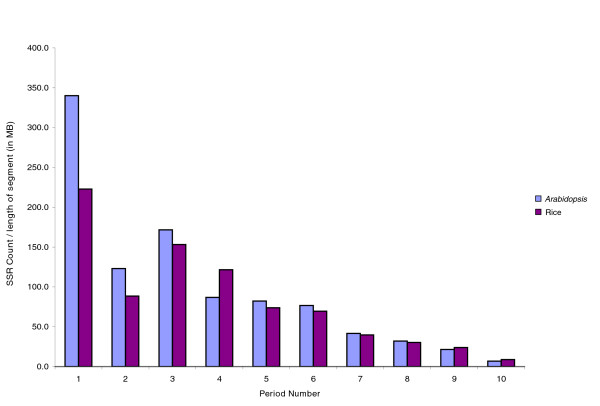
Comparison of 3'UTR SSR densities. A comparison of the SSR densities (for SSRs of period 1 to 10) in the 3'-UTR regions of *Arabidopsis *and rice.

In comparison, the rice 3'UTR regions contain a total of 5,658 SSRs (Table 1). The average SSR density is approximately 832/MB. SSRs with periods of 1 to 10 account for 26.8% (1,515), 10.6% (602), 18.4% (1,042), 14.6% (827), 8.9% (502), 8.3% (472), 4.8% (270), 3.6% (206), 2.9% (162), and 1.1% (60), respectively. Figure 5 shows the corresponding SSR densities.

### Observed versus expected densities of SSRs in different regions

The expected numbers of mono-, di-, tri-, and tetranucleotide SSRs were calculated using the de Wachter formula, as detailed in the methods section. Table 4 shows the observed and expected densities for exon, 5'UTR, 3'-UTR, and intron regions. For both *Arabidopsis *and rice, the 5'UTR, 3'UTR, and intron regions show similar patterns: the observed densities of mono-, di-, tri-, and tetranucleotide SSRs are much higher than those expected. In contrast, for the exon regions, in *Arabidopsis*, only trinucleotide SSRs are more abundant than expected, all other types of SSRs (mono-, di-, and tetranucleotides) being present at a much lower frequency than expected; in rice, the observed densities of all types of SSRs except tetranucleotide SSRs are higher than those expected.

### GO categories of genes with most repeats

In *Arabidopsis*, the average amount of repeats per gene is approximately two. The ten most common Gene Ontology (GO) categories for the genes with high SSR densities are: chloroplast (GO ID: 0009507), nucleus (GO ID: 0005634), mitochondria (GO ID: 0005739), extracellular region (GO ID: 0005576), transcription factor activity (GO ID: 0003700), nucleotide binding (GO ID: 0005524), DNA binding (GO ID: 0003677), structural constituent of cell wall (GO ID: 0005199), cell wall (GO ID: 0005618), and hydrolase activity (GO ID: 0004553). The hypergeometric tests show that there is no statistically significant enrichment of SSRs in genes belonging to these GO categories (*P* > 0.05).

In rice, the average amount of repeats per gene is approximately three. The 10 most common GO categories for the genes with high SSR densities are: transferase activity (GO ID: 0016740), hydrolase activity (GO ID: 0016787), catalytic activity (GO ID: 0003824), response to stress (GO ID: 0006950), membrane (GO ID: 0016020), protein binding (GO ID: 0005515), binding (GO ID: 0005488), DNA binding (GO ID: 0003677), response to biotic stimulus (GO ID: 0009607), and kinase activity (GO ID: 0016301). Among these GO categories, the hypergeometric tests show that SSRs are significantly enriched in genes with GO categories of DNA binding (*P *= 1.93e^-71^), response to stress (*P *= 2.2e^-48^), and binding (*P *= 4.47e^-46^).

### Amino acid runs in coding regions

In coding regions, trinucleotide repeats are in fact amino acid runs. Because each amino acid is encoded by one or more synonymous codon, we are interested in how trinucleotide SSRs have contributed to the single amino acid runs. Specifically, we denote the amino acid runs as 'homogeneous runs' if they are trinucleotide repeats, in which case only one codon is used for the amino acid runs. We created a perl script that we used to analyze the proteomes of *Arabidopsis *and rice and calculated the amounts of amino acid runs of length ≥5 [15,16].

A total of 7,258 amino acid runs (we require at least 5 of the same amino acid in a row) were found in the 26,416 protein sequences in *Arabidopsis*. The five most frequent types of amino acid run are (Table 5): serine with 1,997 runs (27.5%); proline with 865 runs (11.9%); glycine with 853 runs (11.8%); glutamic acid with 831 runs (11.4%); and glutamine with 451 runs (6.2%).

A total of 28,367 amino acid runs were found in the 57,915 protein sequences in rice. The five most frequent types of amino acid run are (Table 5): alanine with 7,477 runs (26.4%); glycine with 6,349 runs (22.4%); proline with 3,727 runs (13.1%); serine with 2,862 runs (10.1%); and arginine with 1,636 (5.8%).

As expected, for both *Arabidopsis *and rice, the proportion of homogeneous runs decreases as the number of synonymous codons increases. Interestingly, we found that the proportions of homogeneous runs in rice are always much higher than that in *Arabidopsis *for all amino acids except aspartic acid and asparagine (Table 5).

The difference in the distribution of amino acid runs could be due to the fact that different amounts of genes from rice and *Arabidopsis *were analyzed. To examine this issue, we analyzed the amino acid runs for only the orthologous genes between rice and *Arabidopsis*. The orthologous genes were downloaded from the Gramene website [17]. The complete list of genes is included in Additional data file 1. Altogether we analyzed 10,519 pairs of orthologous genes and found that the results yielded similar values, with the most frequent types of amino acid runs remaining the same and the proportions staying consistent with the results for all genes (Table 6).

## Discussion

Monocots and dicots are thought to have diverged from a common species approximately 200 million years ago [18]. *Arabidopsis *and rice are the representative species in their respective groups whose genomes, because of their small sizes, have been largely sequenced. *Arabidopsis *has been traditionally used as a model plant species, and rice has gathered much attention due to its significance in being one of the major food resources in the world.

Comparative analyses of the *Arabidopsis *and rice genomes have yielded a number of insights about the two plants. First, since *Arabidopsis *and rice have 5 and 12 chromosomes, respectively, it has been commonly thought that monocots have undergone genome duplication after the split of the monocot and dicot species [18]. However, studies of both the *Arabidopsis *and rice genomes suggest a different story: *Arabidopsis*, despite having the smallest genome among the dicots, might have gone through several rounds of genome duplication [19-21]. In contrast, the rice genome shows no distinct pattern of genome duplication, instead appearing to be more the product of gradual small scale duplications and loss of duplicated genes [22-24].

Second, the *Arabidopsis *genome is compact, with an average gene size of approximately 2.4 kb [22]. In contrast, the rice genes are on average four times larger (approximately 9.9 kb) [25]. The much larger average gene size in rice seems to be due to the larger introns [22,25]. Third, the gene sets in the *Arabidopsis *and rice genomes appear highly asymmetric to each other: approximately 80% to 90% of the *Arabidopsis *genes have rice homologs, yet only 49.4% to 71% of the rice genes have *Arabidopsis *homologs [22,23,25]; therefore, many genes in the rice genome might be monocot specific. In fact, these genes also do not have homologs in other sequenced genomes, including *Drosophila melanogaster*, *Caenorhabditis elegans*, *Saccharomyces cerevisiae*, and *Schizosaccharomyces pombe *[22,23]. Unfortunately, most of these annotated rice genes have no known functions. The question remains as to how so many rice or monocot specific genes have come into existence and what their functional and evolutionary significance is.

Fourth, the G+C content of the *Arabidopsis *genes is rather homogenous; in contrast, the G+C content of the rice genes decreases from the 5'UTR to the 3'UTR by several percent to approximately 25% [22,26]. This gradient of G+C content in rice genes is still present when comparing rice genes with the corresponding orthologs in *Arabidopsis *[22,26]. Here, we further examined the nucleotide components in different regions of the *Arabidopsis *and rice genes. The genome average G+C content is 36% in *Arabidopsis *and 43.6% in rice, and the higher average G+C content in rice than in *Arabidopsis *is consistently observed for all other genic regions (5'UTR, 3'UTR, introns, and exons). In rice, the average G+C content ranking is 5'UTR (55.7%) > exons (53.2%) > introns (43.8%) > 3'UTR (40.2%). In *Arabidopsis*, the G+C content ranking is exons (44.2%) > 5'UTR (38.3%) > 3'UTR (33.8%) > introns (32.5%).

The gradient of G+C content along genes has also been observed in other monocots [26]. This suggests two likely evolutionary scenarios. One is that the ancestral species of monocots and dicots had no G+C gradient along genes and the genome wide G+C incline in monocots formed at early stages after the divergence of monocots and dicots, since otherwise we would have to assume that many monocot species evolved this trait independently. The second scenario is that the ancestral species of monocots and dicots possessed this trait and the dicot species has lost this trait subsequently. One can examine this issue using a proper outgroup species. It remains an open question as to what evolutionary transitions correspond to this genomic trait, if it was acquired in monocots, and the significance of having distinct G+C content in different genic regions.

Fifth, the evolution of SSRs, which is examined in this study in detail, shows several similarities and differences between *Arabidopsis *and rice. In the following, we discuss the similarities and differences both within and between the two genomes.

**Figure 6:**
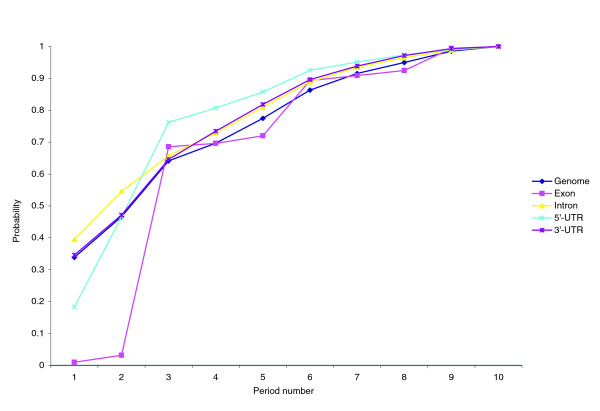
*Arabidopsis *cumulative SSR distribution. Graph showing the cumulative distribution of SSR percentages for periods 1 to 10 in *Arabidopsis*.

**Figure 7:**
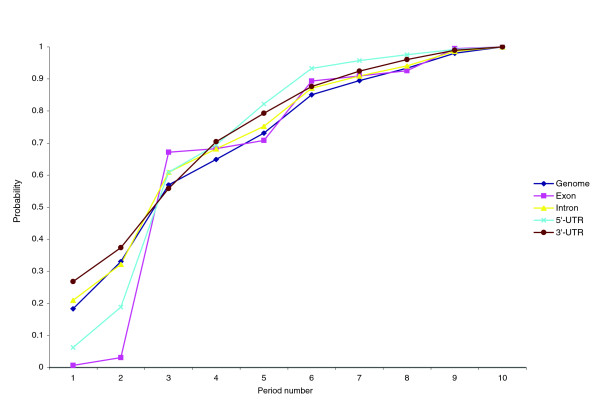
Rice cumulative SSR distribution. Graph showing the cumulative distribution of SSR percentages for periods 1 to 10 in rice.

The results on the SSR distribution show that for both species, the majority of the SSRs are mono-, di-, tri-, tetra-, and pentanucleotides, accounting for up to approximately 80% of all the SSRs found in various regions and the genomes (Figure 6 and 7). For both species, the distribution of SSRs in the 5'-UTRs and exons show patterns distinct from the other genic regions and the entire genomes. Introns and 3'-UTRs have a similar SSR distribution to the whole genome for SSRs with periods of 1 to 10. The discrepancies between *Arabidopsis *and rice SSR distribution are most pronounced for SSRs with periods of 1 to 4 (Figures 1 to 5).

**Figure 8:**
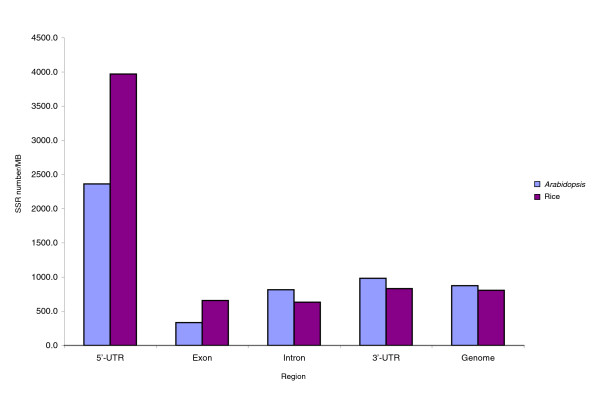
Comparison of SSR densities in different regions. A comparison of the SSR densities across various genic regions.

Remarkably, the average SSR density (measured by the count of SSRs/MB) among all regions for both *Arabidopsis *and rice is highest for the 5'UTRs (Figure 8). A comparison between *Arabidopsis *and rice shows that the average repeat densities in the two genomes is similar for introns, the 3'UTR, and the whole genome, but not for exons and the 5'UTR. The SSR densities in the 5'UTR and exon regions show an almost twofold difference between the two plants, which is the combined result of much higher densities for SSRs of period 3 to 6 in the 5'UTR and much higher densities for SSRs of period 3 and period 6 in the exon regions in rice than in *Arabidopsis *(Figures 2 and 4). Taken together, the 5'UTR and exons stand out as the regions that differ from the remaining regions, a pattern unnoticed before, and deserve further studies on the role of SSRs in them.

In most regions and when doing a comparison of the whole genomes, *Arabidopsis *shows a great affinity for mononucleotide repeats compared to rice (Figures 1 to 5). The comparison between the whole genomes of rice and *Arabidopsis *clearly shows that *Arabidopsis *has a higher percentage of mononucleotide repeats (33.9%) than rice (18.3%). The only regions where mononucleotide repeats are not as high are the 5'UTR and coding regions. Rice seems to have a greater affinity for trinucleotide repeats, with an exceptionally high density of approximately 1,670/MB in the 5'UTR, even higher than in exon regions (approximately 421.4/MB). In fact, trinucleotide SSRs are the major type of SSR in all regions except 3'UTRs (Figures 1 to 5).

Yet despite these differences in which type of SSR is most common for each organism, rice and *Arabidopsis *show similar distribution of the SSR types (period) in their coding regions. Both have a majority of trinucleotide repeats (*Arabidopsis *65.4%, rice 64%) and a lack of any other types other than those divisible by three (the latter account for only 10.4% in *Arabidopsis*, and 10.4% in rice). The high percentage of SSRs with period divisible by three is expected because of the nature of translation and how it relies on triplet codons. It also corresponds with previous research that has shown that tri- and hexanucleotide repeats are the most common in the coding regions of eukaryotes [27,28]. The rather similar distribution of SSRs of period 1 to 10 in the coding regions of the two genomes could be partially explained by the observation that approximately 80% to 90% of the predicted *Arabidopsis *proteins show homology with the predicted rice proteins [22,23,25].

Apart from the similar distribution of the SSRs in coding regions, the two plants show at least two major differences. First, the densities for the tri- and hexanucleotide SSRs are much higher in rice (trinucleotide, approximately 421.4/MB; hexanucleotide, approximately 122.1/MB) than in *Arabidopsis *(trinucleotide, approximately 218.7/MB; hexanucleotide, 57.7/MB). Second, when comparing the amino acid runs, while both plants contain many runs of glycine and proline (accounting for either the second or third highest amounts), they differ in what amino acid occurs in the highest amount of runs. Serine is in the highest amount for *Arabidopsis *and alanine is in the highest amount for rice. However, rice still has a large amount of serine repeats (the fourth largest amount of runs when comparing all of the amino acid runs), while *Arabidopsis *has very few alanine runs. Our initial hypothesis was that this seems to be consistent with the observation that *Arabidopsis *shows homology to rice but rice does not show as much homology to *Arabidopsis *[22,26]. However, we observed the same patterns when we limited our analysis to only orthologous genes between rice and *Arabidopsis*, suggesting that the difference in coding regions between rice and *Arabidopsis *in amino acid runs is not the result of differences in gene content.

Over its long history, *Arabidopsis *has undergone at least three polyploidy events [19], leaving it with many duplicates throughout its genome. In fact, over 37% of genes are part of gene families that contain more than five members. Genes that have duplicates more often than chance are involved in signal transduction and transcription, especially in the nucleus and plasma membrane [29]. According to the data we have analyzed, these types of genes also contain an abundance of SSRs.

In terms of SSR types, a few trends can be observed. Both the rice and *Arabidopsis *genomes have A/T as the most frequent mononucleotide repeats, and the most common dinucleotide repeats are similar between the two genomes as well. However, the most common tri- and tetranucleotide SSR types are mostly different between the two species. What is observable in *Arabidopsis *is that most of the larger SSRs (≥3) consist of long strings of either A or T broken by an additional nucleotide (such as AAAAG or TTTTTC). This shows again a tendency towards mononucleotide repeats. In rice, there is a trend in the trinucleotide repeats which, with little exception, consist of various combinations of C and G. Common SSR types consist of two Cs and one G or two Cs and one T in various combinations (Table 3).

## Conclusion

The SSRs show distinct patterns of distribution among different regions of the genes in both *Arabidopsis *and rice genomes. The amounts of differences in the SSRs between the two genomes are the combined results of ancient species divergences and the individual evolution of these plants. Considering the big discrepancy in gene content between the two plants [22,23], we found the differences between SSRs in *Arabidopsis *and rice less surprising, because of their much higher mutations rates than regular genes [1]. However, the potential functional significance of the SSR changes is an important issue that is yet to be determined.

## Materials and methods

We downloaded the data for *Arabidopsis *from the TAIR website [30] and the data for rice from the TIGR website [31]. For both species, the sequences have already been curated based on their genic locations, including the *Arabidopsis *and rice complete genomic sequences, coding regions (exons of the genes), introns, 5'UTR, 3'UTR, and protein sequences.

We applied **mreps **to search for SSRs [32]; **mreps **is a tool that was specifically developed to identify repeats in DNA sequences. The algorithm consists of a combinatorial and a heuristic treatment to determine SSRs. In the combinatorial step, the maximum runs of tandem repeats are found within the given error threshold. Then in the heuristic treatment, the best candidate SSR is determined for each run and overlapping repeats are merged. The results are also filtered to account for statistically expected results. Afterwards, these results are gathered and iterated for all resolution values until the final resolution value is reached.

We considered only the perfect SSRs with a length of longer than 10 bp. Throughout the paper, we have used the convention of **mreps **and refer to the size of a repeat unit as 'period'. For example, mononucleotide SSRs are SSRs of period 1. Because our analyses revealed that simple repeats with periods greater than 10 are rare, we focused on SSRs with periods 1 to 10. Note that a common and arbitrary definition of SSRs is simple repeats with periods of 1 to 6.

Using perl scripts, we sorted the **mreps **data into files where each SSR was organized by the locus in which it was contained. To examine how the observed numbers of SSRs compared to the expected numbers of SSRs in different genic regions, we calculated the expected number of SSRs using the following formula [33]:

*N*(*M*_*t*_) = *p*(*M*)^*t*^[1 - *p*(*M*)][*N*'(1 - *p*(*M*) + 2*L*]

*N*' = *N *- *tL *- 2*L *+ 1

In this formula, *M *is the repeat unit (repeat type), *N*(*M*_*t*_) is the expected number of times that, in a DNA segment of length *N*, we find *t *consecutive *M*s, *L *is the length of *M*, and *p*(*M*) is the probability of *M *(obtained by multiplying the probability of each nucleotide contained within the repeat unit *M*).

To examine whether SSRs are associated with gene function, we grouped all genes based on their GO [34] categories. The GO data show in what category of protein the gene product of each gene falls. Each gene can belong to multiple categories and these categories are organized into three main categories: molecular function, biological process, and cellular component. Every gene has at least one subcategory from these three main categories assigned to it, with some having additional subcategories as well. For *Arabidopsis*, we downloaded a complete listing of the GO data from the TAIR website. For rice, we downloaded a complete listing of the GO data from the TIGR website. We applied hypergeometric tests to formally examine whether any particular gene functions (GO categories) have statistically significant SSR enrichment [35]. Suppose that we have a total of n genes, among which there are m genes that have high SSR densities. Suppose further that there are r genes that are in a given GO category, of which k genes are in the high-SSR-density class. The following hypergeometric test gives the significance of enrichment of SSRs for this specific GO category:



## Additional data files

The following additional data are available with the online version of this paper. Additional data file 1 contains a list of the orthologous genes that were analyzed.

## Supplementary Material

Additional File 1 A complete list of genes analyzed from rice and *Arabidopsis*. Click here for file

## Figures and Tables

**Table 1:** Total lengths of the studied regions and the amounts of SSRs therein

	Number of base pairs	Number of SSRs	Density (SSRs/MB)
***Arabidopsis***			
5'UTR	260,1047	6,146	2,363.8
Exon	36,447,605	12,168	334.3
Intron	21,826,773	17,756	814.5
3'UTR	5,007,008	4,910	982.0
Genome	118,997,677	104,102	874.8
			
**Rice**			
5'UTR	3,147,158	12,310	3,971.0
Exon	84,149,445	55,338	658.0
Intron	138,050,564	87,529	633.8
3'UTR	6,833,063	5,658	832.1
Genome	370,522,132	298,819	807.4

**Table 2:** Densities of the most abundant SSR (mono- and dinucleotide) types in different regions

	Mononucleotide*	Dinucleotide^†^
***Arabidopsis***		
5'UTR	A: 432.7 (99.6%)	AG: 593.1 (89.3%)
	C: 1.9 (0.4%)	AC: 48.8 (7.4%)
Exons	A: 3.2 (95.9%)	AG: 6.4 (88.3%)
	C: 0.1 (4.1%)	AT: 0.5 (7.2%)
Introns	A: 320.3 (99.6%)	AT: 53.9 (43.9%)
	C: 1.3 (0.4%)	AG: 42.9 (35%)
3'UTR	A: 339 (99.7%)	AT: 52.4 (42.6%)
	C: 1 (0.3%)	AG: 48.2 (39.2%)
Genome	A: 292.6 (98.8%)	AT: 55.9 (50.1%)
	C: 3.7 (1.2%)	AG: 42.6 (38.2%)
		
**Rice**		
5'UTR	A: 182.9 (73.7%)	AG: 380 (75.9%)
	C: 65.2 (26.3%)	AT: 60.3 (12%)
Exons	C: 2.3 (55.4%)	AT: 8.1 (50.1%)
	A: 1.9 (44.6%)	AG: 7.3 (45.2%)
Introns	A: 116.8 (87.9%)	AG: 32.3 (45.6%)
	C: 16.1 (12.1%)	AT: 22 (31.1%)
3'UTR	A: 213.4 (95.8%)	AT: 34.9 (39.4%)
	C: 9.4 (4.2%)	AG: 28.5 (32.2%)
Genome	A: 127.7 (86.2%)	AG: 51.8 (43.6%)
	C: 20.4 (13.8%)	AT: 42.6 (35.9%)

Each of the repeat types contains all circular permutations of not only the sequence in question, but also of the complement of the sequence. For example, 'AG' represents 'AG', 'GA', 'CT', and 'TC'. The unit is per mega-base pairs. The percentage indicates how much percent of all repeats of this period are of this type. *Two possible permutations; ^†^four possible permuations.

**Table 3:** Densities of the most abundant SSR (tri- and tetranucleotide) types in different regions

	Trinucleotide*	Tetranucleotide^†^
***Arabidopsis***		
5'UTR	AAG: 521.9 (74.3%)	AAAG: 41.9 (39.6%)
	AAC: 48.1 (6.8%)	AAAC: 15.8 (14.9%)
Exons	AAG: 78.2 (35.8%)	AAAG: 1 (28.9%)
	AGT: 27.7 (12.6%)	AAAC: 0.7 (19.8%)
Introns	AAG: 35.6 (39.1%)	AAAC: 13.4 (22.8%)
	AAC: 23.1 (25.4%)	AAAG: 12.9 (22%)
3'UTR	AAG: 60.4 (35.2%)	AAAG: 23.4 (27%)
	AAC: 33.2 (19.3%)	AAAC: 21 (24.2%)
Genome	AAG: 64.3 (42%)	AAAT: 15.6 (32.5%)
	AAC: 18.7 (12.2%)	AAAG: 9.3 (19.2%)
		
**Rice**		
5'UTR	CCG: 731.3 (43.8%)	CGAT: 70.6 (20.9%)
	CCT: 401.3 (24%)	CCCT: 37.4 (11.1%)
Exons	CCG: 218.4 (51.8%)	CCCT: 1.1 (14.3%)
	CCT: 58.2 (13.8%)	CCCG: 0.8 (10.7%)
Introns	CCG: 74.2 (40.7%)	AAAT: 5.3 (11.4%)
	CCT: 25.5 (14%)	CGAT: 3.9 (8.5%)
3'UTR	AAG: 23.5 (15.4%)	CGAT: 18.8 (15.5%)
	CCG: 22.4 (14.6%)	AATT: 12.5 (10.3%)
Genome	CCG: 86.3 (44.7%)	CGAT: 7.6 (11.9%)
	AAG: 25.1 (13%)	AAAT: 5.9 (9.2%)

Each of the repeat types contains all circular permutations of not only the sequence in question, but also of the complement of the sequence. The unit is per mega-base pairs. The percentage indicates how much percent of all repeats of this period are of this type. *Ten possible permutations; ^†^32 possible permuations.

**Table 4:** Observed and expected densities of SSRs in different genic regions

	5'-UTR	Exon	Intron	3'-UTR
***Arabidopsis***				
Mononucleotide	434.6 (12.3)	3.3 (4.8)	321.6 (65.7)	33.9 (3.2)
Dinucleotide	664.2 (20.4)	7.3 (12.9)	122.7 (33.9)	12.3 (3.1)
Trinucleotide	702.7 (7.7)	218.7 (4.5)	90.9 (17.6)	17.1 (1.4)
Tetranucleotide	105.8 (27.7)	3.3 (17.9)	58.7 (58.9)	8.7 (4.5)
				
**Rice**				
Mononucleotide	248.1 (6.1)	4.2 (3.7)	132.9 (4.9)	222.8 (11.5)
Dinucleotide	500.6 (13.2)	16.1 (11.5)	70.9 (12.8)	88.5 (16.9)
Trinucleotide	1670.6 (4.5)	421.4 (3.9)	182.3 (4.4)	153.2 (6.5)
Tetranucleotide	337.4 (18.7)	7.6 (16.1)	46.2 (17.7)	121.6 (24.3)

The unit is per mega-base pairs. The numbers listed in parentheses are the expected densities of various periods in different regions.

**Table 5:** Numbers of amino acid runs and homogeneous runs

Amino acid*	*Arabidopsis*			Rice		
	
	Number of amino acid runs	Number of H-runs^†^	% H-runs^‡^	Number of amino acid runs	Number of H-runs^†^	% H-runs^‡^
Ala (4)	327	43	13.1	7,477	3,318	44.4
Gly (4)	853	171	20.0	6,349	2,461	38.8
Pro (4)	865	132	15.3	3,727	1,221	32.8
Ser (6)	1,997	322	16.1	2,862	742	25.9
Arg (6)	103	9	8.7	1,636	311	19.0
Glu (2)	831	370	44.5	1,387	734	52.9
Asp (2)	424	246	58.0	1,269	697	54.9
Gln (2)	451	169	37.5	1,077	634	58.9
Leu (6)	275	20	7.3	882	286	32.4
Thr (4)	215	64	29.8	479	264	55.1
Lys (2)	394	183	46.4	314	174	55.4
His (2)	146	87	59.6	303	218	71.9
Val (4)	51	5	9.8	267	108	40.4
Asn (2)	247	137	55.5	249	49	19.7
Phe (2)	46	28	60.9	34	24	70.6
Cys (2)	3	1	33.3	22	16	72.7
Met (1)	18	18	100.0	13	13	100.0
Trp (1)	0	0	0.0	7	7	100.0
Tyr (2)	4	0	0.0	7	6	85.7
Ile (3)	8	2	25.0	6	2	33.3

*Numbers in parentheses indicate the numbers of codons that code for the amino acid. ^†^'H-runs' refers to amino acid runs that consist of the exact same codon, equivalent to trinucleotide SSRs. ^‡^'% H-runs' is the percentage of homogeneous runs.

**Table 6:** Distribution of amino acid runs of only the orthologous genes between *Arabidopsis *and rice, in comparison with the total genes in the two species

Amino acid	*Arabidopsis*				Rice			
	
	Runs*	% runs^†^	O-runs^‡^	% O-runs^§^	Runs*	% runs^†^	O-runs^‡^	% O-runs^§^
Ala	327	4.5	188	5.2	7,477	26.4	2,572	31.1
Gly	853	11.8	397	10.9	6,349	22.4	1,868	22.6
Pro	865	11.9	342	9.4	3,727	13.1	999	12.1
Ser	1,997	27.5	946	26.1	2,862	10.1	861	10.4
Arg	103	1.4	43	1.2	1,636	5.8	395	4.8
Glu	831	11.4	421	11.6	1,387	4.9	314	3.8
Asp	424	5.8	208	5.7	1,269	4.5	311	3.8
Gln	451	6.2	421	11.6	1,077	3.8	249	3.0
Leu	275	3.8	122	3.4	882	3.1	212	2.6
Thr	215	3.0	110	3.0	479	1.7	148	1.8
Lys	394	5.4	174	4.8	314	1.1	126	1.5
His	146	2.0	83	2.3	303	1.1	104	1.3
Val	51	0.7	20	0.6	267	0.9	62	0.8
Asn	247	3.4	127	3.5	249	0.9	16	0.2
Phe	46	0.6	18	0.5	34	0.1	15	0.2
Cys	3	0.0	2	0.1	22	0.1	7	0.1
Met	18	0.2	6	0.2	13	0.0	3	0.0
Trp	4	0.1	2	0.1	7	0.0	2	0.0
Tyr	0	0.0	0	0.0	7	0.0	1	0.0
Ile	8	0.1	1	0.0	6	0.0	1	0.0
Total	7,258	100	3,631	100	28,367	100	8,266	100

*The number of amino acid runs found in all *Arabidopsis *or rice genes. ^†^The percentages of individual types of amino acid runs. ^‡^'O-runs' refers to the number of amino acid runs found in only the orthologous genes between *Arabidopsis *and rice. ^§^'% O-runs' is the percentage of individual types of amino acid runs for the orthologous genes between *Arabidopsis *and rice.
